# Comparative Study of a Novel Lateral Flow Rapid Test with Conventional Serological Test Systems for the Diagnosis of Canine Leishmaniosis in Croatia and Brazil

**DOI:** 10.3390/pathogens13020109

**Published:** 2024-01-26

**Authors:** Rouzbeh Mahdavi, Franjo Martinkovic, Hosam Shams-Eldin, Ingrid E. Pereira, Alexandre B. Reis, Andreas Latz, Daniela Heinz, Cristina Aira, Alba Fresco-Taboada, Elfadil Abass, Jelena Romero-Olmedo, Henrique C. Teixeira, Ulrich Steinhoff

**Affiliations:** 1 Institute of Medical Microbiology and Hospital Hygiene, Phillips University of Marburg, 35043 Marburg, Germany; mahdavir@staff.uni-marburg.de (R.M.); shamseld@staff.uni-marburg.de (H.S.-E.); jelena.romero.olmedo@uniklinik-freiburg.de (J.R.-O.); 2 Faculty of Veterinary Medicine, University of Zagreb, HR-1000 Zagreb, Croatia; fmartinkovic@gmail.com; 3 Department of Parasitology, Microbiology and Immunology, Federal University of Juiz de Fora, Juiz de Fora 36036-900, MG, Brazilhenrique.teixeira@ufjf.br (H.C.T.); 4 School of Pharmacy, Federal University of Ouro Preto, Ouro Preto 35400-000, MG, Brazil; alexreis@nupeb.ufop.br; 5 Gold Standard Diagnostics Frankfurt (GSD Frankfurt), 63128 Dietzenbach, Germany; andreas.latz@eu.goldstandarddiagnostics.com (A.L.); daniela.heinz@eu.goldstandarddiagnostics.com (D.H.); 6 Gold Standard Diagnostics Madrid S.A. (GSD Madrid), 28037 Madrid, Spain; cristina.airapino@eu.goldstandarddiagnostics.com (C.A.); alba.fresco@eu.goldstandarddiagnostics.com (A.F.-T.); 7 Department of Clinical Laboratory Science, College of Applied Medical Sciences, Imam Abdulrahman Bin Faisal University, Dammam 34212, Saudi Arabia; emabass@iau.edu.sa

**Keywords:** canine leishmaniosis, CanL, POC diagnostics for leishmaniosis, lateral flow test, line blot, *Leishmania-*improved serodiagnostics, *Leishmania* ELISA

## Abstract

Control of canine infections with *Leishmania infantum* (*L. infantum*), a major zoonotic disease in Brazil and southern Europe, is becoming increasingly important due to its close proximity to humans, the increasing import of dogs from endemic regions and the impact of climate change on vector spreading. Simple, rapid and reliable diagnostic tests are therefore needed to detect infected dogs. Here, we re-evaluated different serological methods for the diagnosis of canine leishmaniosis (CanL) in Croatia and Brazil. The diagnostic performance of the indirect fluorescent antibody test (IFAT) and the VetLine^®^ Leishmania ELISA (GSD Frankfurt, Germany) was compared with three rKLi8.3-based diagnostic test systems, the rKLi8.3 ELISA (GSD Frankfurt, Germany), the INgezim^®^ Leishma CROM (GSD Madrid, Spain) lateral flow test (LFT) and the VetBlot^®^ *Leishmania* LineBlot (GSD Frankfurt, Germany). CanL symptomatic dogs were efficiently diagnosed by all tests, except the VetLine^®^ Leishmania ELISA, which is based on whole *Leishmania* antigens. The advantage of rKLi8.3 was also observed in oligo- and asymptomatic dogs from Brazil and Croatia, although with reduced diagnostic efficiency compared to symptomatic dogs. Similar to IFAT and rKLi8.3 ELISA, the LFT did not cross-react with other common canine pathogens; it showed very high specificity for healthy dogs from endemic regions in both countries and did not react with healthy, vaccinated dogs in Brazil. In conclusion, serodiagnostic tests based on the rKLi8.3 antigens are superior to whole parasite antigens, and the LFT has the advantage of providing a laboratory-independent, rapid and specific diagnosis of CanL.

## 1. Introduction

Leishmanioses are diseases caused by protozoan flagellate parasites of the genus *Leishmania.* Some of them are causative agents of disease in humans, dogs, cats, horses, rodents, etc., but most of them, 20 out of 30 species are zoonotic [1]. The life cycle of *Leishmania* parasites is dixenous, i.e., it includes the vertebrate—and arthropod—host although they may be transmitted directly from the vertebrate host without the arthropod vector [2,3,4,5].

In vertebrates, *Leishmania* sp. parasitizes intracellularly in mononuclear phagocytes as amastigote forms while in the biological vector, e.g., sandflies of the genera *Phlebotomus* and *Lutzomyia*, they live extracellularly as promastigote forms [6]. Infections with *Leishmania* parasites occur mainly in tropical and poor areas of Africa, Asia and Latin America, but also increasingly in countries of southern Europe. They can cause cutaneous (CL) or visceral leishmaniasis (VL) in humans and canine leishmaniosis (CanL) in dogs [7,8].

From the aspect of veterinary medicine, CanL is most important, although the disease can be diagnosed in other domestic and wild animals [9]. Dogs are important reservoirs of the *Leishmania* parasites, i.e., the causative agent of CanL and human leishmaniasis, which in Europe is caused by the most prevalent species—*L. infantum* [10], while in Brazil the disease has been related to eight species of *Leishmania*, mainly *L. infantum* and *L. brasiliensis* [1]. Although wild animals may be involved in the transmission of *Leishmania*, dogs are the most relevant reservoir in urban areas due to their close association with humans [11]. The zoonotic nature of *L. infantum* is a serious concern for animal and public health. In the Americas, Brazil is the country responsible for the endemic disease of leishmaniasis, with 96% of the VL cases occurring in this country. Although surveys that assess the VL/CanL situation in Brazil are scarce, the expansion of cities into forest areas, the high number of domestic dogs and the adaptability of sandflies are likely to be responsible for the spread of leishmaniasis in northeastern municipalities of Brazil [12,13,14]. Thus, dogs have become the main target for disease control [15]. In Europe, the import of *L. infantum* into previously non-endemic countries has been known for a long time, but lately, it has become a problem and seems to be a consequence of the increased travelling with dogs into endemic areas and the import of infected puppies and stray dogs from endemic areas [16,17,18,19]. Furthermore, global warming favors the occurrence of the sandfly vector in these countries increasing the risk of transmission [20]. Animal and human health authorities have recognized the emergence of leishmaniasis in part of the European Union as a serious public health problem and have stressed the importance of surveillance, notification and control of this disease [10].

CanL shows a variable, non-specific spectrum of clinical signs depending on the immune status, and infected dogs are often asymptomatic, which is a major challenge as they may contribute to the spread of the disease despite the lack of clinical signs; therefore, detection of such animals is important but remains mostly incidental [21]. Here we used a classification of four clinical stages based on clinical signs, clinicopathological abnormalities and serological status. This was proposed by the LeishVet group in an attempt to cover the wide range of clinical manifestations and degrees of severity seen in CanL [22] and has been further developed for the consensus recommendation [23].

The serological tests already available such as the indirect fluorescent antibody test (IFAT), ELISA, lineblot and lateral flow tests (LFT) play a central role in disease surveillance because they are inexpensive, and in particular, the LFT is easy to use [24]. However, they often fail to detect subclinical infections, may cross-react with other infectious agents and do not always discriminate infected from vaccinated dogs, as many of these tests are based on whole *Leishmania* antigens [25,26]. To ameliorate the serodiagnosis of leishmaniosis, we recently developed a new recombinant kinesin antigen from *L. infantum* (rKLi8.3) that has shown improved diagnostic performance in VL patients, independent of their endemic area origin [27].

The aim of this study was to compare the diagnostic performance of different serodiagnostic methods on a panel of sera from Croatian and Brazilian dogs that have been classified as *Leishmania*-positive or negative dogs by IFAT, parasitological examination and/or Dual Path Platform test (DPP^®^, Fiocruz, Manguinhos, Rio de Janeiro, Brasil). The results demonstrate that ELISA, lineblot and LFT based on the rKLi8.3 antigen exhibit superior diagnostic performance with regard to symptomatic (SD), oligosymptomatic (OD), asymptomatic (AD), vaccinated dogs and those suffering from other canine infections.

## 2. Materials and Methods

### 2.1. Canine Serum Sample

We analyzed serum samples from 232 Croatian and 112 Brazilian adult dogs of both sexes. Croatian samples were obtained from a serum repository of the Serological Animal Laboratory, Department for Parasitology and Invasive Diseases with Clinics, Faculty of Veterinary Medicine, University of Zagreb and included asymptomatic and symptomatic animals with serologically proven leishmaniosis, healthy controls and sera from dogs with other infections.

Brazilian samples were obtained from a serum repository of the Laboratory of Immunopathology at the Federal University of Ouro Preto, Minas Gerais and comprised sera from parasitological confirmed (*L. infantum*), asymptomatic, oligosymptomatic, symptomatic dogs and non-infected, endemic controls (EC). VAC (*n* = 20) sera from healthy dogs immunized with the Leish-Tec^®^ vaccine (Ceva Hertape AS, Juatuba, Brazil) were received from the blood bank of the Santo Agostinho Veterinary Hospital, Belo Horizonte, Minas Gerais, Brazil (Supplementary Materials Table S1).

### 2.2. Clinical Examinations

Dogs positive for CanL were classified according to the presence of clinical symptoms into three groups: asymptomatic (AD; *n* = 11), without clinical suggestive signs of the disease; oligosymptomatic (OD; *n* = 12), with a maximum of three symptoms indicative of CanL including dull hair and/or localized alopecia and/or moderate weight loss; symptomatic (SD; *n* = 13), with clinical signs characteristic of CanL, such as dull hair, severe weight loss, onychogryphosis, skin lesions, apathy and keratoconjunctivitis [28].

### 2.3. Serological Tests

IFAT-classified sera were re-evaluated by two ELISAs and one lateral flow test (LFT): VetLine^®^ Leishmania ELISA test (GSD Frankfurt, Germany) based on native *L. infantum* antigens, recombinant KLi8.3 antigen-based ELISA (rKLi8.3 ELISA, GSD Frankfurt, Germany) and the rKLi8.3 LFT (INgezim^®^ Leishma CROM, GSD Madrid, Spain) [27]. For ELISAs, samples were probed in duplicates, and optical densities (OD) were read in a spectrophotometer. The mean was used to classify samples as positive, negative or ambiguous (uncertain), following kit instructions. The principle of the LFT is shown in Supplementary Materials Figure S1, and tests were performed according to the manufacturer’s instructions. Briefly, 10 μL of serum was added to the sample window of the test device, followed by 4 drops of buffer provided in the kits. The test was read 10 min after the addition of the buffer. The results were positive if two distinct red or pink lines appeared (test and control region), negative when the control region was positive but the test region negative and invalid if the control line failed to appear. LFT was performed twice, and critical sera (low antibody titers/asymptomatic dogs) were analyzed in triplicate.

For IFAT, the antigen was prepared from promastigotes of *L. infantum* MON-1 (archive isolate from continuous in vitro culture—MCAN/HR/2011/SO) for Croatia and *L. amazonensis* (MHOM/BR/1960/BH6) and *L. chagasi* (MHOM/BR/1972/BH46) for Brazil, and anti-*Leishmania* antibodies were detected using goat or rabbit anti-dog FITC IgG. Cytoplasmic or membranous green fluorescence of promastigotes scored positive with a cutoff dilution of 1/40. As controls, CanL-positive and -negative sera were included [29].

Recombinant antigens, rK28, rK39 and rLb6H, were produced at the Infectious Disease Research Institute (IDRI), Seattle, USA. ELISAs with these antigens were performed as previously described [30].

For lineblots (VetBlot^®^ *Leishmania* LineBlot, GSD Frankfurt, Germany), recombinant rKLi8.3, rK39 and rKLO8 and a native *Leishmania* antigen were printed with a dispenser (FrontLine HR microliter contact; BioDot, Irvine, CA, USA) on a nitrocellulose membrane (GE Healthcare, Chicago, IL, USA), together with a control line for sample loading and for conjugate function as shown in Supplementary Materials Figure S2. After drying, the membranes were cut into 3 mm stripes and stored at 4 °C until use. Prior to use, stripes were equilibrated in 1 mL of sample dilution buffer (10 mM phosphate buffer, pH 7.2). Samples were added in a dilution of 1:100 and the membranes were incubated with gentle shaking for 1 h at room temperature. After 3 washes with 1 mL washing buffer (0.2 M phosphate, pH = 7.2) for 5 min each, the membranes were incubated with gentle shaking for 30 min with 1 mL horseradish peroxidase (HRP)-labeled protein A/G conjugate at room temperature. The stripes were washed three times with 1 mL of washing buffer for 5 min. The development of the signals took place by incubation of the membranes with 1 mL of 3,3′,5,5′-tetramethylbenzidine (TMB) substrate solution for 15 min with gentle shaking at room temperature. The reaction was stopped by the addition of at least 1 mL of distilled water. After drying the membranes for at least 30 min at room temperature, the stripes were evaluated.

### 2.4. Statistical Analyses

Statistical analysis of the mean optical densities (ODs) of infected and healthy groups was performed using the Mann–Whitney U test. Comparisons of the mean ODs from asymptomatic, oligosymptomatic, symptomatic and healthy dogs, as well as dogs with other diseases, were performed using the Kruskal–Wallis test, followed by Dunn’s Multiple Comparison Test (GraphPad Software 9.0, San Diego, CA, USA). Sensitivity and specificity were calculated with their 95% confidence intervals. *p* values of <0.05 were considered statistically significant.

## 3. Results

### 3.1. Comparative Testing of IFAT, ELISA and LFT for CanL Serodiagnosis in Croatia

IFAT-characterized sera from Croatian dogs were re-evaluated by the VetLine^®^ ELISA, the rKLi8.3 ELISA and the LFT.

The rKLi8.3-based ELISA and LFT showed the highest diagnostic performance. Of 66 symptomatic dogs, 64 animals were positive in both, rKLi8.3 ELISA and LFT. This corresponds to a sensitivity of 96.9%. IFAT detected 63 and VetLine^®^ ELISA 58 dogs, corresponding to 95.4% and 87.8% sensitivity, respectively. Of the 27 asymptomatic dogs, 19 sera were positive by the VetLine^®^ ELISA, while 23 and 22 dogs were positive by the rKLi8.3 ELISA and the LFT, respectively. Interestingly, 24 animals were CanL-positive by IFAT, corresponding to 88.8% sensitivity.

To assess the specificity of these tests, we analyzed 88 serum samples from healthy dogs originating from the same endemic area. The specificity was as follows: IFAT (98.8%), VetLine^®^ ELISA (85.2%), rKLi8.3 ELISA (98.8%) and LFT (97.7%) (Table 1).

Furthermore, potential cross-reactivity of the test systems was assessed with 51 serum samples from dogs that were parasitological positive for *Canine babesiosis*, *Giardia duodenalis*, *Dirofilaria repens*, *Toxocara canis*, *Ehrlichia canis*, *Ancylostoma caninum* and *Anaplasmosis*. While the VetLine^®^ ELISA cross-reacted with 2 sera of canine babesiosis, none of the 51 sera cross-reacted with IFAT, rKLi8.3 ELISA and LFT.

**Figure 1:**
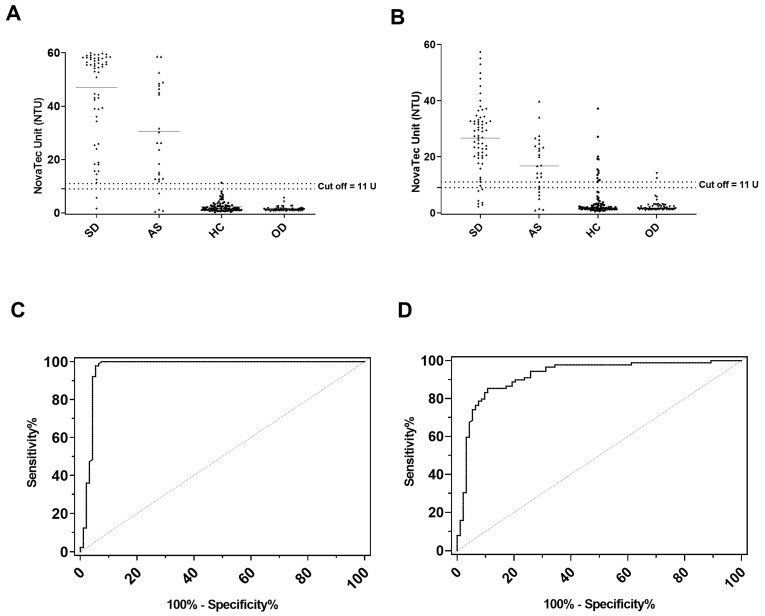
CanL-specific antibody responses in canine sera from Croatia were measured by (**A**) rKLi8.3 ELISA and (**B**) VetLine^®^ ELISA. The manufacturer-defined borderline zone is indicated by dotted lines; data within were considered negative. Roc curve analysis of rKLi8.3 (**C**) and VetLine^®^ELISA (**D**). AS = asymptomatic; HC = healthy controls; OD = other diseases.

Comparison of the ELISAs indicated that rKLi8.3 ELISA showed the highest area under the curve (AUC), with a value of 0.9664 and a confidence interval (CI) 95%: 0.9342 to 0.9986, whereas VetLine^®^ ELISA showed an AUC of 0.9221, CI 95%: 0.8805 to 0.9638 (Figure 1).

### 3.2. Evaluation of the rKLi8.3 ELISA and LFT in Healthy, Vaccinated and Infected Dogs from Brazil

**Figure 2:**
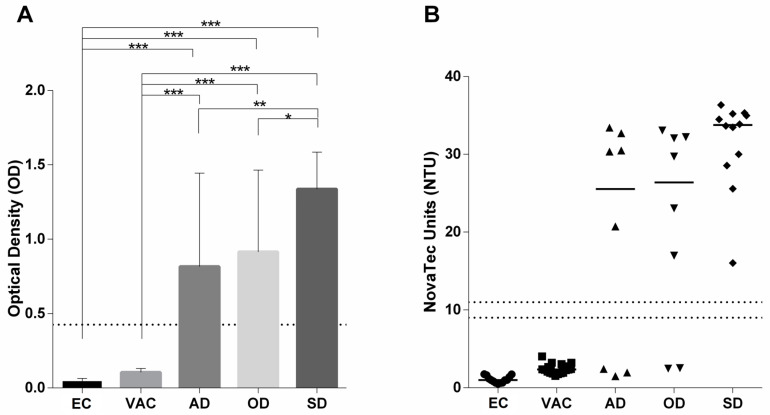
CanL-specific antibody responses in dog sera from Brazil were measured by rKLi8.3 ELISA and shown as (**A**) optical densities or (**B**) as individual dot blots in NovaTec units, according to the manufacturer. The borderline zone is indicated by dotted lines. AD = asymptomatic dogs; OD = oligosymptomatic dogs; SD = symptomatic dogs; VAC = vaccinated dogs; EC = healthy endemic controls. * *p* = 0.1; ** *p* = 0.01; *** *p* = 0.001.

We next analyzed a cohort of Brazilian dogs that comprised parasitologically confirmed cases of CanL, including asymptomatic (AD), oligosymptomatic (OD) and symptomatic (SD) infected dogs. As controls, non-vaccinated (EC) and vaccinated (Leish-Tec^®^) healthy dogs (VAC) from the same endemic area were investigated by the rKLi8.3 ELISA and LFT. All SD (12/12), 6/8 OD and 5/8 AD dogs were positive in ELISA as well as LFT, and none of the healthy and vaccinated control animals showed any signals in ELISA (Figure 2 and Supplementary Materials Table S2). It is interesting to note that both rKLi8.3-based tests, ELISA and LFT, gave identical results in all investigated groups. Accordingly, the sensitivity of the ELISA and LFT for SD, OD and AD was 100%, 75% and 62%, respectively, and their specificity for VAC and EC was 100%. Of note, rKLi8.3-based ELISA and LFT did not cross-react with autochtonous *T. cruzi*-infected dogs from Brazil (Supplementary Materials Figure S3, Table S3).

ELISAs using recombinant rK28, rK39 and rLb6H antigens were also performed on the same serum panel. With the rK28 antigen, 13/13 SD, 9/12 OD and 8/11 AD were identified as positive. The corresponding sensitivities are 100%, 75% and 72.7%. For the rK39 antigen, 12/13 SD, 10/12 OD and 8/11 AD were positive, representing sensitivities of 92.3%, 83.3% and 72.7%. With the rLb6H antigen, 13/13 SD, 11/12 OD and 11/11 AD were positive, giving a sensitivity of 100% for SD and AD and 90.9% for OD. The rK28 exhibited 100% specificity, rK39 91.6% and rLb6H only 14.2% (Supplementary Materials Figure S4).

### 3.3. Detection of CanL-Specific IgG Antibodies in Dogs from Croatia and Brazil by rKLi8.3 Lineblot

About 24 sera from SD, 15 from AD and 52 from EC dogs from Croatia, previously tested positive or negative for CanL by IFAT, VetLine^®^ ELISA, rKLi8.3 ELISA and LFT INgezim^®^, were also tested with the rKLi8.3 lineblot (Supplementary Materials Table S4).

In SD, all the above-mentioned test systems showed 100% sensitivity. For AD, rKLi8.3 lineblot showed 80%, rKLi8.3 ELISA 86.6%, LFT INgezim^®^ 86.6% and VetLine^®^ ELISA 66.6% sensitivity. The EC group exhibited 94.2% specificity with the rKLi8.3 lineblot, 100% with the rKLi8.3 ELISA, 98% with the LFT and 86.5% with the VetLine^®^ ELISA (Table 2).

Furthermore, we analyzed Brazilian dogs with parasitologically confirmed CanL cases, including AD, OD and SD dogs by lineblot. EC and vaccinated healthy dogs were used as controls (Supplementary Materials Table S5). In the SD group (*n* = 13), all sera were positive, and in the AD group (*n* = 11), 6 sera were positive, showing a sensitivity of 54.5%. In the OD group (*n* = 11), 8 sera were positive, exhibiting a sensitivity of 72.7%. All sera from the EC groups (*n* = 13) and vaccinated dogs (*n* = 16) were tested negative, showing 100% specificity.

## 4. Discussion

*Leishmania*-infected dogs act as the main reservoir for the zoonotic disease and play a crucial role in the transmission to humans. Increased travel and the import of dogs by animal welfare organizations bear the risk of spreading leishmaniosis to countries where it has not previously occurred. Part of the problem is also that surveillance and notification of canine leishmaniosis has a low priority among animal health authorities [10,31].

To minimize the risk of spreading infections with *L. infantum*, systematic infection control of dogs originating from or moved to endemic areas is required. Thus, there is a need for a simple and reliable, point-of-care test. The currently used test systems, e.g., ELISA, IFAT, PCR and parasitological examinations, require a laboratory and are not suitable for a fast and simple routine diagnostic [32]. Furthermore, some of the diagnostic tests show low specificity and cross-reactivity between *Leishmania* sp. and other canine pathogens, such as *Ehrlichia*, *Babesia canis*, *Toxoplasma gondii* and *T. cruzi*, or are unable to differentiate between vaccinated and infected dogs [30,33].

An additional challenge of CanL diagnosis is infected but clinically asymptomatic dogs because they represent a potential reservoir of the parasite [21]. The detection of asymptomatic dogs often represents a problem, as *Leishmania*-specific antibody levels are either very low or some animals never become seropositive or revert to seronegative while they still harbor the parasite [34,35,36,37].

IFAT is most commonly used to diagnose CanL in Mediterranean countries [34,38]. However, IFAT is a complex and time-consuming technique that relies on the experience of the observer, as positive antigen–antibody reactions are assessed by the fluorescence intensity. It needs great expertise, and results may vary between different investigators. In our study, IFAT was highly sensitive for SD and AD and showed high specificity in EC, while other studies reported sensitivities ranging from 68% to 100% and specificities from 60% to 90% [39,40].

Commercially available ELISAs that are based on whole parasite antigens often show low reproducibility due to the use of different *Leishmania* species and thus variant antigen compositions that react with different VL antibodies [41].

Furthermore, several recombinant antigens, such as rK39, rK26, rK28, rLb6H and rKLO8, have been used for serodiagnosis in humans and dogs [27,37,42,43,44]. Although the reported sensitivities of these antigens are generally high for symptomatic dogs, variable sensitivities (98–64%) have been described for asymptomatic dogs [34,37,44]. These discrepancies illustrate the problems of comparing different studies, especially when carried out on different canine serum samples.

The rKLi8.3 kinesin antigen is characterized by a high content of charged amino acids from *L. infantum* and an increased number of B-cell epitopes. This antigen demonstrated superior diagnostic efficacy over rK39 in diagnosing human VL when used in the ELISA and LFT format [27]; however, its diagnostic potential in dogs has not been investigated.

The CanL screening of 232 Croatian dogs revealed that both, rKLi8.3 ELISA and LFT, have a very similar diagnostic sensitivity with regard to symptomatic (both 96.9%) and asymptomatic (85.1%; 81.4%) dogs. Furthermore, both tests showed 100% specificity for animals with other infections and about 98% when tested in healthy endemic controls. Similar results were also observed for the rKLi8.3 lineblot. It is interesting to note that the IFAT results were comparable to rKLi8.3-based test systems, with the difference that IFAT was slightly better in AD (88.8%) and marginally less sensitive in SD (93.9%). In contrast, the VetLine^®^ ELISA showed a significantly poorer diagnostic efficiency, with a sensitivity of 87.8% in SD and 70.3% in AD groups.

The extension of the study with Brazilian dogs provided additional insights into the diagnostic performance of the rKLi8.3 based ELISA, lineblot and LFT in a different endemic area.

Screening of 72 sera from parasitologically tested dogs from Belo Horizonte, Brazil, showed that all three tests gave very similar results and that the performance of these tests was independent of the endemic area. Neither vaccinated nor endemic controls gave positive signals in either test, and rKLi8.3 did not cross-react with sera from *T. cruzi*-infected dogs.

Notably, the same sera (except for vaccinated animals) were tested in ELISA with rK28, rK39 and rLb6H antigens, but none of these antigens outperformed the rKLi8.3 antigen in terms of SD, OD and AD group sensitivities or specificity for EC group.

In conclusion, the study shows that the rKLi8.3-based test systems are highly sensitive and specific diagnostic tools for Leishmaniosis in humans and dogs, irrespective of the endemic area. However, it is important to note that, unlike parasitological methods, serological tests are fundamentally dependent on the amount of *Leishmania*-specific antibodies, which are not present in all asymptomatic animals. However, the high diagnostic performance, combined with simple use, rapid results and laboratory independence, make the rKLi8.3 LFT suitable for routine and large-scale diagnosis in veterinary practices and in the field.

## Figures and Tables

**Table 1:** Diagnostic performance of Commercial VetLine^®^ ELISA, rKLi8.3 ELISA, LFT and IFAT for CanL in Croatia.

Dogs (*n*)	In house IFAT			VetLine^®^ ELISA			rKLi8.3 ELISA			LFT INgezim^®^		
	Pos	Neg		Pos	Neg		Pos	Neg		Pos	Neg	
Symptomatic (66)	63	3	Sensitivity 95.4%	58	8	Sensitivity 87.8%	64	2	Sensitivity 96.9%	64	2	Sensitivity 96.9%
Asymptomatic (27)	24	3	Sensitivity 88.8%	19	8	Sensitivity 70.3%	23	4	Sensitivity 85.1%	22	5	Sensitivity 81.4%
Healthy endemic (88)	1	87	Specificity 98.8%	13	73	Specificity 85.2%	1	87	Specificity 98.8%	2	86	Specificity 97.7%
Other infections (51)	0	51	Specificity 100%	2	49	Specificity 96%	0	51	Specificity 100%	0	51	Specificity 100%

Pos, Positive; Neg, Negative.

**Table 2:** Diagnostic performance of VetLine^®^ ELISA, rKLi8.3 ELISA, LFT and rKLi8.3 lineblot for CanL in Croatia.

Dogs (*n*)	VetBlot Leishmania^®^ LineBlot			VetLine^®^ ELISA			rKLi8.3 ELISA			LFT INgezim^®^		
	Pos	Neg		Pos	Neg		Pos	Neg		Pos	Neg	
Symptomatic (24)	24	0	Sensitivity 100%	24	0	Sensitivity 100%	24	0	Sensitivity 100%	24	0	Sensitivity 100%
Asymptomatic (15)	12	3	Sensitivity 80%	10	5	Sensitivity 66.6%	13	2	Sensitivity 86.6%	13	2	Sensitivity 86.6%
Healthy endemic (52)	3	49	Specificity 94.2%	7	45	Specificity 86.5%	0	52	Specificity 100%	1	51	Specificity 98%

Pos, Positive; Neg, Negative.

## Data Availability

Data will be made available upon request.

## Supplementary Materials

The following supporting information can be downloaded at https://www.mdpi.com/article/10.3390/pathogens13020109/s1, Table S1: Panel of canine sera from Croatia and Brazil; Figure S1: Scheme of the lateral flow assay (INgezim^®^ Leishma CROM, Spain) for the detection of antibodies to *Leishmania*; Figure S2: Scheme of the VetBlot^®^ *Leishmania* Lineblot for the detection of CanL antibodies; Table S2: Comparison of CVL seropositivity by rKLi8.3 based LFT and ELISA and ELISAs based on other recombinant antigens; Figure S3: Sera from *T. cruzi* infected dogs with acute and chronic Chagas disease were tested by rKLi8.3 ELISA.; Table S3: Comparison of CVL seropositivity by rKLi8.3 based LFT and -ELISA in acute and chronic *T. cruzi* infected dogs; Figure S4: Comparison of anti-*Leishmania* antibody responses in rK28-rK39- and rLb6H ELISA using symptomatic (SD), oligosymptomatic (OD), asymptomatic (AD) and endemic control sera from Brazil; Table S4: Croatian dog sera tested by lineblot; Table S5: Brazilian dog sera tested by lineblot.
