# Supplementary material for: Comparative Study of a Novel Lateral Flow Rapid Test with Conventional Serological Test Systems for the Diagnosis of Canine Leishmaniosis in Croatia and Brazil
Source: Pathogens. 2024 Jan 26;13(2):109. doi: 10.3390/pathogens13020109 (PMC10892489; doi:10.3390/pathogens13020109)
Supplement: Supplementary file 1 [file pathogens-13-00109-s001.zip › pathogens-2775682-supplementary.pdf]

Supplemental Table S1. Panel of canine sera from Croatia and Brazil that have been pre-examined for CVL by indicated tests and were used for re-evaluation by VetLine® ELISA, rKLi8.3 ELISA and LFT (INgezim® Leishma CROM) tests.

Dogs	Region	Diagnosis	n
**Symptomatic**	Croatia	Leishmania Dipstick Rapydtest, Apacor/IFAT	66
**Asymptomatic**	Croatia	„	27
**Healthy endemic controls**	Croatia	„	88
**Other infection**	Croatia	parasitological examination	51
**Symptomatic**	Brazil	parasitological examination/ DPP®	13
**Oligosymptomatic**	Brazil	parasitological examination/ DPP®	12
**Asymptomatic**	Brazil	parasitological examination/ DPP®	11
**Healthy endemic controls**	Brazil	parasitological examination/ DPP®	16
**Healthy vaccinated**	Brazil	parasitological examination/ DPP®	20
***T. cruzi* infected (Chagas)**	Brazil	parasitological examination	40
**Total**			344

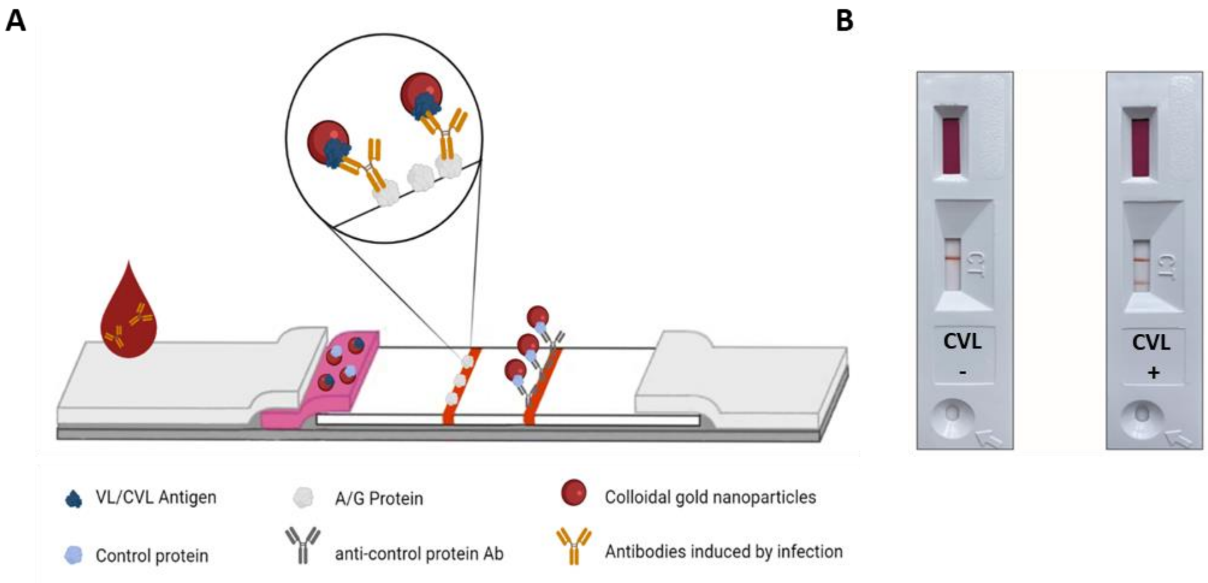

Supplemental Figure S1. A) Scheme of the lateral flow assay (INgezim® Leishma CROM, Spain) for the detection of antibodies to *Leishmania*. B) Representative result of LFT with negative and positive CVL sera.

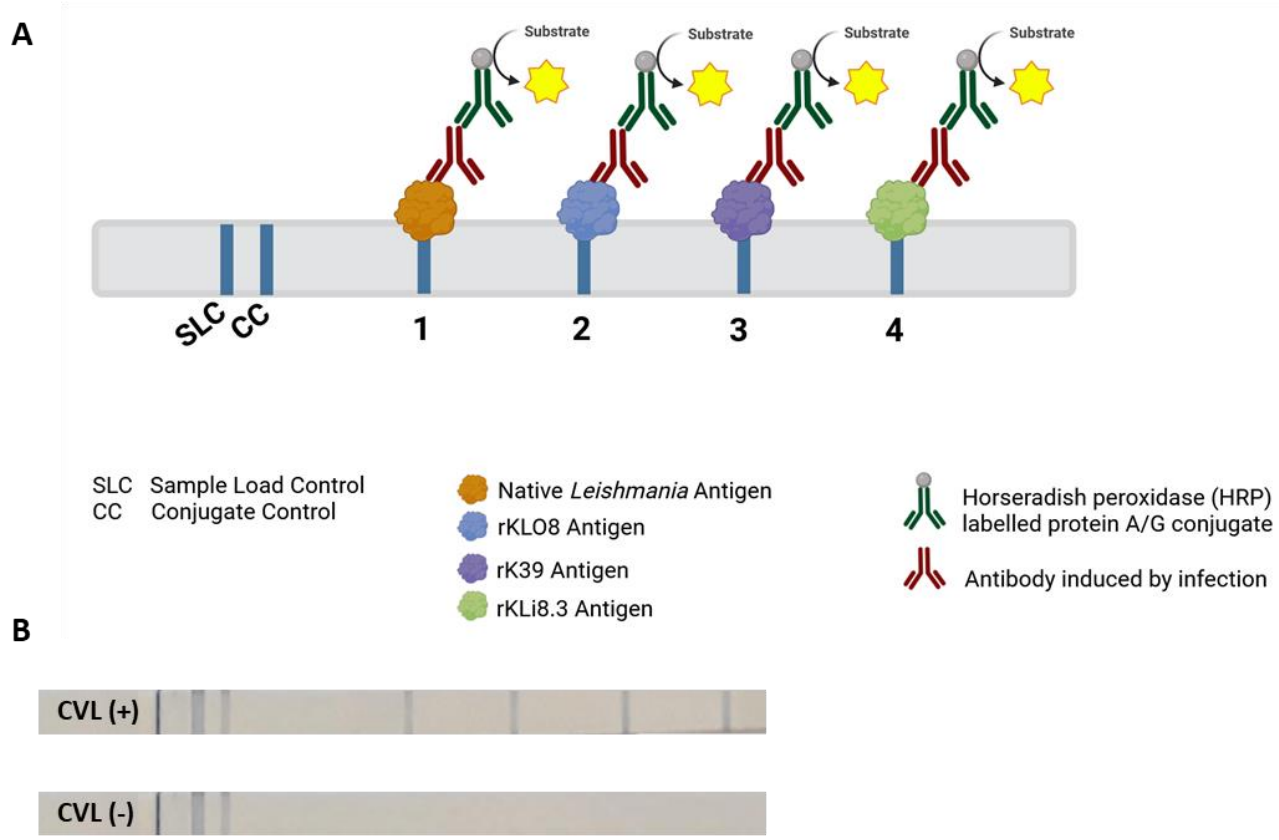

Supplemental Figure S2. A) Scheme of the VetBlot® Leishmania Lineblot for the detection of CanL antibodies. Line 1, native *Leishmania* antigen and line 2-4, recombinant antigens of *Leishmania* are printed onto a nitrocellulose membrane. B) Representative result of a lineblot with negative and positive CanL sera.

Supplemental Table S2. Comparison of CVL seropositivity by rKLi8.3 based LFT and ELISA and ELISAs based on other recombinant antigens.

	Animals	LFT	rKLi8.3	rK28	rK39
Grupo AD	AD1	-	-	-	-
	AD2	ND	ND	-	+
	AD3	-	-	-	-
	AD4	+	+	+	+
	AD5	-	-	+	-
	AD6	+	+	+	+
	AD7	+	+	+	+
	AD8	ND	ND	+	+
	AD9	+	+	+	+
	AD10	ND	ND	+	+
	AD11	+	+	+	+
Grupo OD	OD2	-	-	-	-
	OD3	+	+	+	+
	OD4	+	+	+	+
	OD5	+	+	+	+
	OD6	+	+	+	+
	OD7	ND	ND	+	+
	OD8	ND	ND	+	+
	OD9	+	+	+	+
	OD10	+	+	+	+
	OD11	ND	-	-	-
	OD12	ND	ND	+	+
Grupo SD	SD1	+	+	+	+
	SD2	+	+	+	+
	SD3	+	+	+	+
	SD4	+	+	+	+
	SD5	+	+	+	+
	SD6	+	+	+	+
	SD7	+	+	+	+
	SD8	+	+	+	+
	SD9	+	+	+	-
	SD10	+	+	+	+
	SD11	ND	ND	+	+
	SD12	+	+	+	+
	SD13	+	+	+	+

IgG seropositivity of dogs divided into asymptomatic (AD), oligosymptomatic (OD) and symptomatic (SD) CVL, based on the cut-off point established for each antigen. Animals whose optical density was greater than the cutoff point were considered positive (+) and those whose optical density was less than or equal to the cutoff point were considered negative (-). LFT = Lateral flow test based on the rKLi8.3 antigen. ND: Not Done.

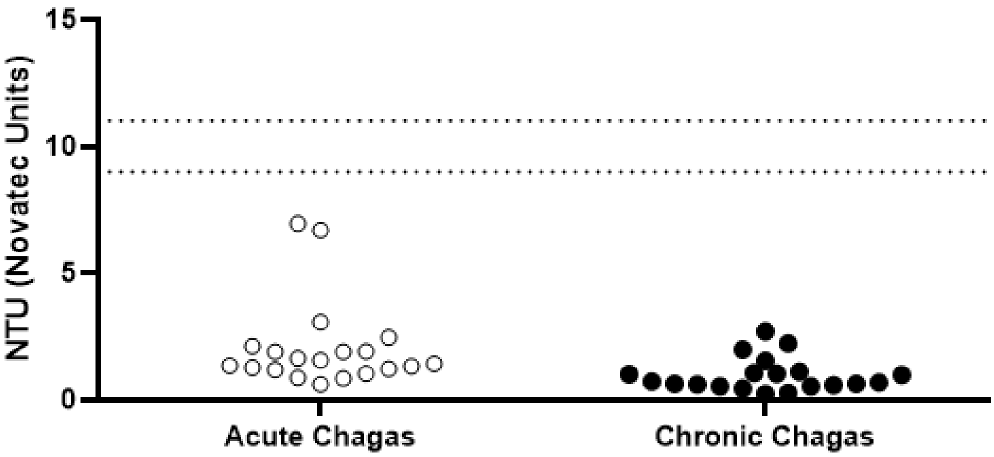

Supplemental Figure S3. Sera from *T. cruzi* infected dogs with acute and chronic Chagas disease were tested by rKLi8.3 ELISA.

Supplemental Table S3. Comparison of CVL seropositivity by rKLi8.3 based LFT and -ELISA in acute and chronic *T.cruzi* infected dogs.

Groups	Animals	LFT	ELISA
Acute Chagas	AC2	-	-
	AC3	-	-
	AC4	-	-
	AC5	-	-
	AC11	-	-
	AC12	-	-
	AC13	-	-
	AC16	-	-
	AC18	-	-
	AC19	-	-
Chronic Chagas	CC1	-	-
	CC6	-	-
	CC7	-	-
	CC8	-	-
	CC9	-	-
	CC10	-	-
	CC14	-	-
	CC15	-	-
	CC17	-	-
	CC20	-	-

IgG seropositivity of dogs divided into acute and chronic Chagas. Animals whose optical density was greater than the cutoff point were considered positive (+) and those whose optical density was less than or equal to the cutoff point were considered negative (-). LFT = Lateral flow test based on the rKLi8.3 antigen.

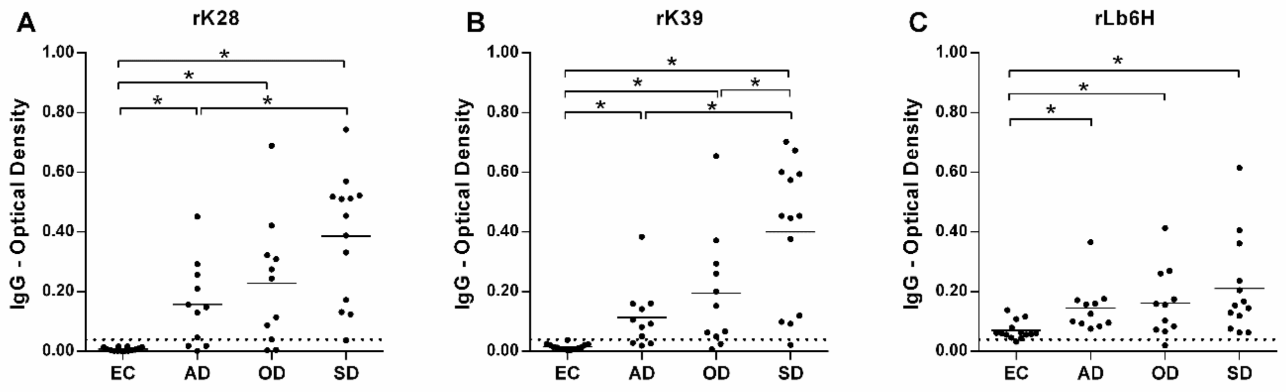

Supplemental Figure S4. Comparison of anti-Leishmania antibody responses in rK28- rK39- and rLb6H ELISA using symptomatic (SD), oligosymptomatic (OD), asymptomatic (AD) and endemic control sera from Brazil.

Supplemental Table S4. Croatian dog sera tested by lineblot*:* reactivity with individual antigens are shown**.**

Groups	Animals	(native, rKLO8, rK39, rKLi8.3)
SD	**3280**	1+, 2+, 3+, 4+
	**3285**	1+, 2+, 3+, 4+
	**3286**	1+, 2+, 3+, 4+
	**3288**	1+, 2+, 3+, 4+
	**3292**	1+, 2+, 3+, 4+
	**3293**	1+, 2+, 3+, 4+
	**3297**	1+, 2+, 3+, 4+
	**3298**	1+, 2+, 3+, 4+
	**3299**	1+, 2+, 3+, 4+
	**3302**	1+, 2+, 3+, 4+
	**3303**	1+, 2+, 3+, 4+
	**3304**	1+, 2+, 3+, 4+
	**3308**	1+, 2+, 3+, 4+
	**3309**	1+, 2+, 3+, 4+
	**3310**	1+, 2+, 3+, 4+
	**3334**	1+, 2+, 3+, 4+
	**3341**	1+, 2+, 3+, 4+
	**3347**	1+, 2+, 3+, 4+
	**3348**	1+, 2+, 3+, 4+
	**3357**	1+, 2+, 3+, 4+
	**3358**	1+, 2+, 3+, 4+
	**3361**	1+, 2+, 3+, 4+
	**3365**	1+, 2+, 3+, 4+
	**3371**	1+, 2+, 3+,4+
AD	**3254**	1-, 2-, 3-, 4-
	**3289**	1+, 2+, 3+, 4+
	**3307**	1+, 2+, 3+, 4+
	**3312**	1+, 2+, 3+, 4+
	**3336**	1+, 2+, 3+, 4+
	**3337**	1+, 2+, 3+, 4+
	**3338**	1+, 2+, 3+, 4+
	**3354**	1w+,2+,3+,4+
	**3355**	1+, 2+, 3+, 4+
	**3356**	1+, 2+, 3+, 4+
	**3364**	1+, 2w+, 3+, 4+
	**3368**	1w+, 2+, 3+, 4+
	**3373**	1w+, 2-, 3-, 4-
	**3375**	1-, 2-, 3-, 4-
	**3377**	1w+, 2w+, 3+, 4+
EC	**3262**	1w+, 2-, 3-, 4-
	**3278**	1w+, 2-, 3-, 4-
	**3279**	1w+, 2-, 3-, 4-
	**3281**	1w+, 2-, 3-, 4-
	**3282**	1-, 2-, 3-, 4-
	**3283**	1-, 2-, 3-, 4-
	**3284**	1-, 2-, 3-, 4-
	**3287**	1-, 2-, 3-, 4-
	**3290**	1w+, 2-, 3-, 4-
	**3296**	1w+, 2-, 3-, 4-
	**3300**	1w+, 2-, 3-, 4-
	**3301**	1+, 2-, 3-, 4-
	**3305**	1w+, 2-, 3-, 4-
	**3306**	1w+, 2-, 3-, 4-
	**3311**	1w+, 2-, 3-, 4-
	**3313**	1-, 2-, 3-, 4-
	**3314**	1-, 2-, 3-, 4-
	**3315**	1w+, 2-, 3-, 4-
	**3322**	1w+, 2-, 3-, 4-
	**3323**	1-, 2-, 3-, 4-
	**3331**	1-, 2-, 3-, 4-
	**3332**	1w+, 2-, 3-, 4-

Groups	Animals	(native, rKLO8, rK39, rKLi8.3)
EC	**3335**	1-, 2-, 3-, 4-
	**3339**	1-, 2-, 3-, 4-
	**3340**	1w+, 2-, 3-, 4-
	**3343**	1-, 2-, 3-, 4-
	**3346**	1-, 2-, 3-, 4-
	**3349**	1-, 2-, 3-, 4-
	**3350**	1-, 2-, 3-, 4-
	**3353**	1-, 2-, 3-, 4-
	**3359**	1w+,2-,3-,4-
	**3360**	1-, 2-, 3-, 4-
	**3362**	1+, 2w+, 3+, 4+
	**3363**	1-, 2-, 3-, 4-
	**3366**	1-, 2-, 3-, 4-
	**3367**	1-, 2-, 3-, 4-
	**3369**	1+, 2-, 3-, 4-
	**3372**	1w+, 2-, 3-, 4-
	**3374**	1+, 2-, 3-, 4-
	**3376**	1+, 2-, 3-, 4-
	**3378**	1+, 2-, 3+, 4w+
	**3379**	1w+, 2-, 3-, 4-
	**3380**	1w+, 2-, 3w+, 4-
	**3381**	1w+, 2-, 3-, 4-
	**3382**	1+, 2-, 3+, 4w+
	**3294**	1w+, 2-, 3-, 4-
	**3295**	1w+, 2-, 3-, 4-
	**3317**	1-, 2-, 3-, 4-
	**3318**	1-, 2-, 3-, 4-
	**3320**	1w+, 2-, 3-, 4-
	**3344**	1w+, 2-, 3-, 4-
	**3345**	1w+, 2-, 3-, 4-

Supplemental Table S5. Brazilian dog sera tested by lineblot: reactivity with individual antigens are shown.

Groups	Animals	(native, rKLO8, rK39, rKLi8.3)
SD	SD1	1w+, 2+, 3+, 4+
	SD2	1+, 2+, 3+, 4+
	SD3	1+, 2+, 3+, 4+
	SD4	1+, 2+, 3+, 4+
	SD5	1+, 2+, 3+, 4+
	SD6	1+, 2+, 3+, 4w+
	SD7	1+, 2+, 3+, 4+
	SD8	1+, 2+, 3+, 4+
	SD9	1+, 2+, 3+, 4+
	SD10	1+, 2+, 3+, 4+
	SD12	1+, 2w+, 3+, 4+
	SD13	1+, 2+, 3+, 4+
	SD14	1+, 2+, 3+, 4+
AD	AD1	1-, 2-, 3-, 4-
	AD2	1-, 2-, 3-, 4-
	AD3	1-, 2-, 3-, 4-
	AD4	1+, 2+, 3+, 4+
	AD5	1w+, 2-, 3-, 4-
	AD6	1+, 2+, 3+, 4+
	AD7	1+, 2w+, 3+, 4+
	AD8	1+,2+,3+,4+
	AD9	1+, 2+, 3+, 4+
	AD10	1+, 2w+, 3w+, 4-
	AD11	1+, 2+, 3+, 4+
OD	OD1	1w+, 2-, 3-, 4-
	OD2	1w+, 2-, 3-, 4-
	OD3	1+, 2+, 3+, 4+
	OD4	1+, 2+, 3+, 4+
	OD5	1+, 2+, 3+, 4+
	OD6	1+, 2+, 3+, 4w+
	OD8	1+, 2+, 3+, 4+
	OD9	1+,2+,3+,4+
	OD10	1+, 2+, 3+, 4+
	OD11	1w+, 2-, 3-, 4-
	OD12	1+,2+,3+,4+
EC	SAE1	1-, 2-, 3-, 4-
	SAE2	1-, 2-, 3-, 4-
	SAE3	1-, 2-, 3-, 4-
	SAE4	1-, 2-, 3-, 4-
	SAE8	1-, 2-, 3-, 4-
	SAE9	1-, 2-, 3-, 4-
	SAE10	1-, 2-, 3-, 4-
	SAE11	1-, 2-, 3-, 4-
	SAE12	1-, 2-, 3-, 4-
	SAE13	1-, 2-, 3-, 4-
	SAE14	1-, 2-, 3-, 4-
	SAE16	1-, 2-, 3-, 4-
VAC	LEIVT 01	1-, 2-, 3-, 4-
	LEIVT 02	1-, 2-, 3-, 4-
	LEIVT 03	1-, 2-, 3-, 4-
	LEIVT 04	1-, 2-, 3-, 4-
	LEIVT 05	1-, 2-, 3-, 4-
	LEIVT 05	1-, 2-, 3-, 4-
	LEIVT 06	1-, 2-, 3-, 4-
	LEIVT 07	1-, 2-, 3-, 4-
	LEIVT 08	1-, 2-, 3-, 4-
	LEIVT 09	1-, 2-, 3-, 4-
	LEIVT 10	1-, 2-, 3-, 4-

Groups	Animals	(native, rKLO8, rK39, rKLi8.3)
**VAC**	**LEIVT 12**	1-, 2-, 3-, 4-
	**LEIVT 13**	1-, 2-, 3-, 4-
	**LEIVT 14**	1-, 2-, 3-, 4-
	**LEIVT 15**	1-, 2-, 3-, 4-
	**LEIVT 16**	1-, 2-, 3-, 4-
